# RDAU-Net: Based on a Residual Convolutional Neural Network With DFP and CBAM for Brain Tumor Segmentation

**DOI:** 10.3389/fonc.2022.805263

**Published:** 2022-03-02

**Authors:** Jingjing Wang, Zishu Yu, Zhenye Luan, Jinwen Ren, Yanhua Zhao, Gang Yu

**Affiliations:** ^1^ College of Physics and Electronics Science, Shandong Normal University, Jinan, China; ^2^ Obstetrics and Gynecology, Tengzhou Xigang Central Health Center, Tengzhou, China

**Keywords:** brain tumor segmentation, U-Net, attention mechanism, dilation feature pyramid, deep learning

## Abstract

Due to the high heterogeneity of brain tumors, automatic segmentation of brain tumors remains a challenging task. In this paper, we propose RDAU-Net by adding dilated feature pyramid blocks with 3D CBAM blocks and inserting 3D CBAM blocks after skip-connection layers. Moreover, a CBAM with channel attention and spatial attention facilitates the combination of more expressive feature information, thereby leading to more efficient extraction of contextual information from images of various scales. The performance was evaluated on the Multimodal Brain Tumor Segmentation (BraTS) challenge data. Experimental results show that RDAU-Net achieves state-of-the-art performance. The Dice coefficient for WT on the BraTS 2019 dataset exceeded the baseline value by 9.2%.

## Introduction

Tumors that grow in the skull are commonly referred to as brain tumors and include primary brain tumors, which occur in the brain parenchyma, and secondary brain tumors, which metastasize to the skull from other parts of the body. According to the World Health Organization (WHO) classification criteria, brain tumors are classified into four grades: grade I, astrocytoma; grade II, oligodendroma gliomas; grade III, anaplastic glioma; and grade IV, glioblastoma multiforme (GBM) (1). The lower the grade of the tumor, the less malignant it is, and the better the prognosis is. As a result, early diagnosis of brain tumors is very important for treatment.

Magnetic resonance imaging (MRI) is considered a standard technique due to its satisfactory soft-tissue contrast and wide availability (2). MRI is a noninvasive imaging technique that uses magnetic resonance phenomena to obtain electromagnetic signals from the human body and reconstruct information about the body as a type of tomography. MRI is available in a variety of imaging sequences. These imaging sequences can produce MRI images with distinctive features that can reflect the anatomical morphology of the human body.

In current clinical practice, brain tumors are labeled manually by physicians, which is time-consuming. Moreover, brain tumors are similar to normal brain tissues in terms of morphology and intensity; hence, manual labeling by physicians suffers from subjective variability and lacks reproducibility (3). Therefore, accurate automatic segmentation of brain tumors in T1, T1-c, T2, and FLAIR is essential for quantitative analysis and evaluation of brain tumors (4).

### Progress on Image Segmentation Methods

In recent years, deep neural network (DNN)-based methods have achieved high performance for brain tumor segmentation (5–8). Convolutional neural networks (CNNs) (9) have achieved great success in many research areas, such as image recognition (10–12), image segmentation (13–15), and natural language processing (16, 17). In (18), a fully convolutional neural network (FCN) for image pixel-level image classification was proposed, which solves the problem of semantic-level image segmentation with input data of arbitrary size. Ronneberger et al. (19) proposed the U-Net framework with a skip connection module connecting the encoder and decoder. In contrast to FCNs, U-Net fuses shallow and deep features and has produced impressive results in medical image segmentation. Inspired by U-Net, Attention U-Net (20) and ResU-Net (21) were proposed and used for medical image segmentation. In Attention U-Net, an attention mechanism is added to the skip connection part. This module generates gating information to readjust the weight coefficients of features at various spatial locations. In ResU-Net, each convolutional layer is replaced with a residual convolutional layer, thereby avoiding gradient disappearance in backpropagation in deep network structures.

### Attention Mechanism

Attention mechanisms were first introduced in natural language processing (22–25). Currently, attention mechanisms are also widely used in deep learning to enhance feature extraction (26–28). Hu et al. (29) proposed plug-and-play squeeze-and-excitation (SE) attention, which learns feature relationships to obtain contextual information on channel dimensions by global average pooling. Wang et al. (30) proposed ECANet, which uses a local cross-channel interaction strategy without downscaling and adaptive selection of one-dimensional convolutional kernels. In addition to these single-channel attention mechanisms, there are several dual-attention mechanisms. For example, Fu et al. *(* 31) proposed the dual attention mechanism network (DANet) to improve the accuracy of network segmentation by capturing feature dependencies based on the spatial and channel dimensions of the self-attention mechanism and summing the outputs of the two modules. Woo et al. (32) proposed a convolutional block attention module (CBAM) to enhance useful information and suppress useless information by a tandem channel attention mechanism and a spatial attention mechanism.

Although ResU-Net uses a residual module to mitigate the problem of vanishing network gradients, it still suffers from the following problems: (1) Multiscale features have an important role, but ResU-Net does not extract features from images of various sizes, and thus, a substantial amount of detailed information is lost. (2) The skip connection cascades the shallow features of the decoder part and the corresponding depth features to achieve feature fusion, but the shallow features of the encoder contain considerable redundant information, which, in turn, affects the segmentation results.

In this paper, we propose RADU-Net, which is an improved version of ResU-Net that is inspired by the attention mechanism. Our contributions are mainly as follows.

1. We insert the 3D CBAM dual attention mechanism in each residual module to alleviate the problem of gradient disappearance or explosion as the network structure deepens and obtain the feature information of the image more accurately.
2. We add the dilated feature pyramid module with the 3D CBAM dual attention mechanism between the encoder and the decoder as a solution to the problem that the traditional U-Net network does not extract multi-scale features of images to obtain feature maps of different sizes.
3. We insert a 3D CBAM block after the skip connection in each layer to improve the extraction of channel information and spatial information to reduce the redundant information of low-level features.

Via these modifications, RADU-Net solves the above problems and improves the overall segmentation accuracy of brain tumors.

## Methods

### RDAU-Net

**Figure 1:**
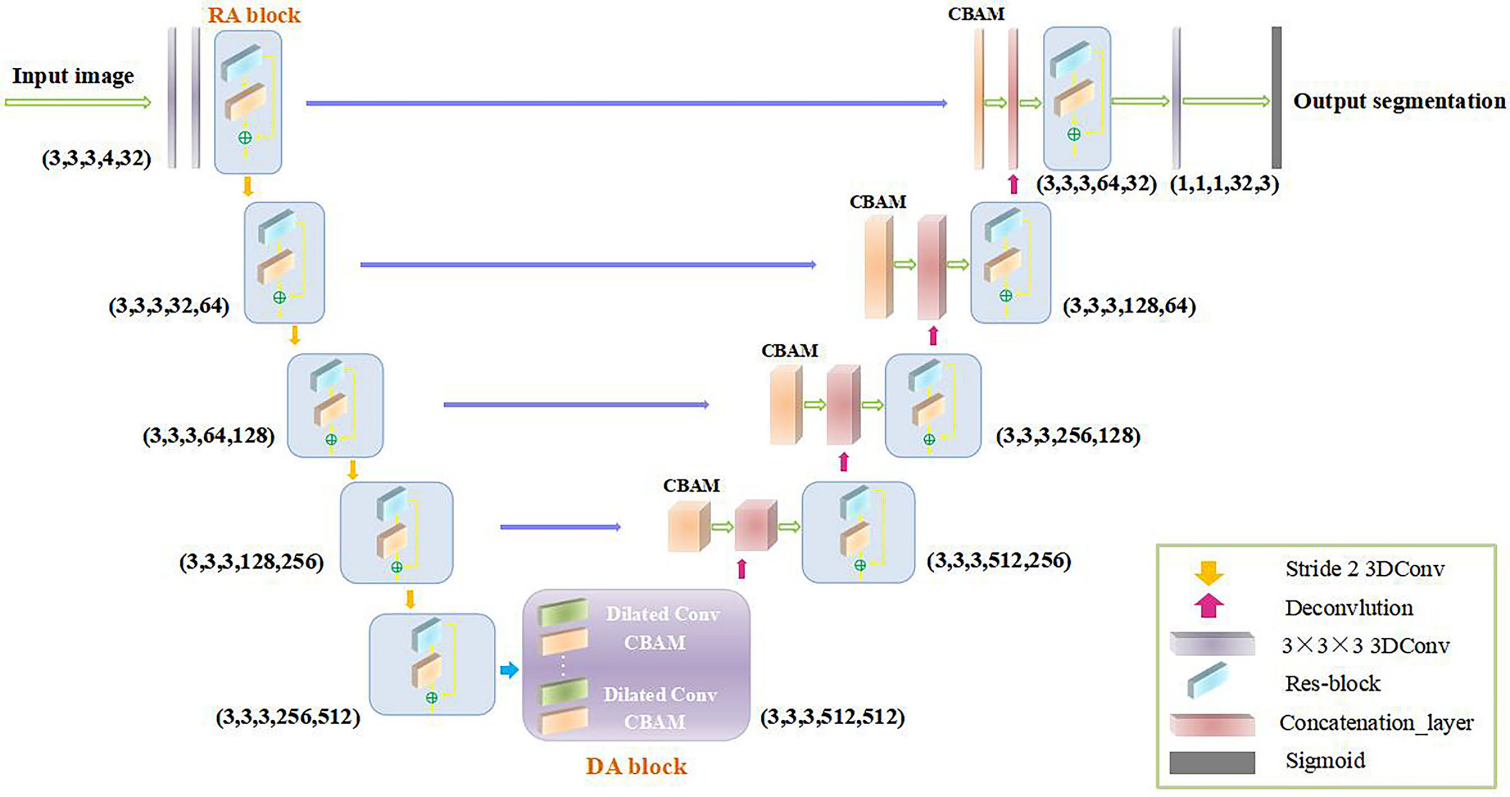
Architecture of the proposed RDAU-Net.

This paper proposes a 3D convolutional neural network, namely, RDAU-Net, for the brain tumor segmentation task. The 3D CNN considers more comprehensive spatial context information and achieves more accurate performance than the 2D CNN in image segmentation. **Figure 1** illustrates the complete structure of RDAU-Net. RDAU-Net includes an encoder part and a decoder part. The input of the encoder part includes 2 purple 3D convolutional layers with a convolutional kernel size of 3 × 3 × 3, 5 3D RA blocks of various sizes and 4 3D convolutional layers with a step size of 2 as the downsampling layer. The RA block is that the orange CBAM block is added after each convolutional layer of the residual block for feature extraction. Between the encoder and the decoder is the DA block, which is the CBAM block that is added after each dilated convolution layer. The decoder part is symmetric to the encoder and contains 4 deconvolution layers. The purple arrow between the encoder and decoder is the skip connection, and a CBAM block is inserted after each skip connection layer. The pink block is the concatenation layer. The network ends with a 3D convolutional layer and a gray block of sigmoid function layers. (3,3,3,4,32) in the first layer indicates that the convolution kernel size of this layer is 3 × 3 × 3, the number of input features is 4 and the number of output features is 32.

### 3D CBAM Block

**Figure 2:**
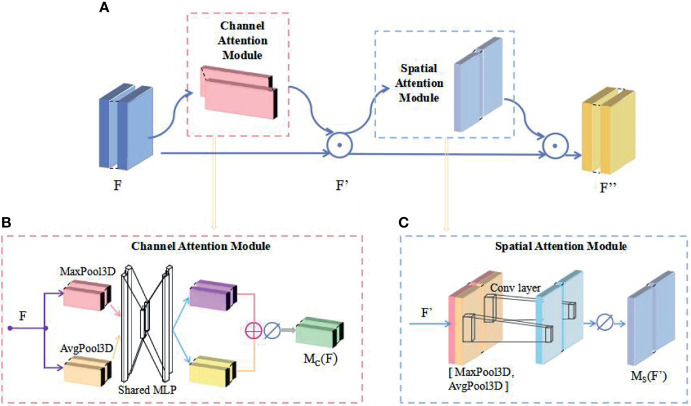
Structure of a 3D CBAM block. **(A)** The overview of 3D CBAM; **(B)** Structure of the Channel Attention Module; **(C)** Structure of the Spatial Attention Module.

To extract more accurate image feature information, we use CBAM (32) as the attention module of the network. We transform the 2D CBAM attention module into a 3D CBAM attention module. CBAM ties together channel attention and spatial attention to increase the weights of useful features in the channel and useful features in the space, as illustrated in **Figure 2A**. First, the deep blue module in **Figure 2A**, which is denoted as *F* ∈ *R^W^* ^×^ *^H^* ^×^ *^D^* ^×^ *^C^*, is input into the channel attention module. The channel attention module as shown in **Figure 2B** consists of a 3D global max pooling module (the pink module in **Figure 2B**), a 3D global average pooling module (the yellow module in **Figure 2B**), and a shared MLP (multilayer perception) that consists of a 3D neural network. The input feature map F is subjected to 3D global max pooling and 3D global average pooling operations and to MLP. The two outputs of MLP are added elementwise. Then, after the sigmoid activation function, we obtain the weight *M_c_* (*F*) after the channel attention module.


(1) $$M_{C}(F)=\sigma (MLP(AvgPool3D(F)+MLP(MaxPool3D(F))=\sigma (W_{1}(W_{0}(F_{avg}^{C})+W_{1}(W_{0}(F_{\max}^{C}))$$

where *W*_0_∈ *R^C^* ^/^ *^r^* ^×^ *^C^*, *W*_1_∈ *R^C^* ^/^ *^C^* ^×^ *^r^*, and σ is the sigmoid activation function. r takes a value of 16, namely, the channel *C* is changed to *C*/16 during max-pooling and average pooling to reduce the number of parameters.

Then, *M_c_* (*F*) is multiplied with the input feature map *F* to obtain the output feature map *F*′ of the channel attention module, and the formula is as follows:


(2) $$F'=M_{C}(F)⊗F$$

where *M_c_* (*F*) denotes the output weight after the channel attention module and ⊗ denotes element-by-element multiplication.

*F*′ is used as the input feature map for the spatial attention model as shown in **Figure 2C**, and the channels are compressed by 3D global max pooling and 3D global average pooling in the channel dimension. The two extracted feature maps $F_{avg}^{S}$ and $F_{\max}^{S}$ are subjected to a channel-based merging operation to obtain a 2-channel feature map, which is subsequently downscaled into a single channel (the shallow blue module in **Figure 2C**) by a 7×7 convolution operation before application of the sigmoid function to generate the output weight *M_S_* (*F*′) in the blue part of the spatial attention module.


(3) $$M_{S}(F”)=\sigma (f^{7\times 7}([AvgPool3D(F”);MaxPool3D(F”)]))=\sigma (f^{7\times 7}([F_{avg}^{S};F_{\max}^{S}]))$$

Finally, *M_S_* (*F*′) is multiplied by *F*′ to obtain the final output feature map *F*′′ of the yellow module, as expressed in Equation (3).


(4) $$F”=M_{S}(F”)⊗F”$$

where *M_S_* (*F*′) denotes the output weight after spatial attention and ⊗ denotes element-by-element multiplication.

### RA Block

**Figure 3:**
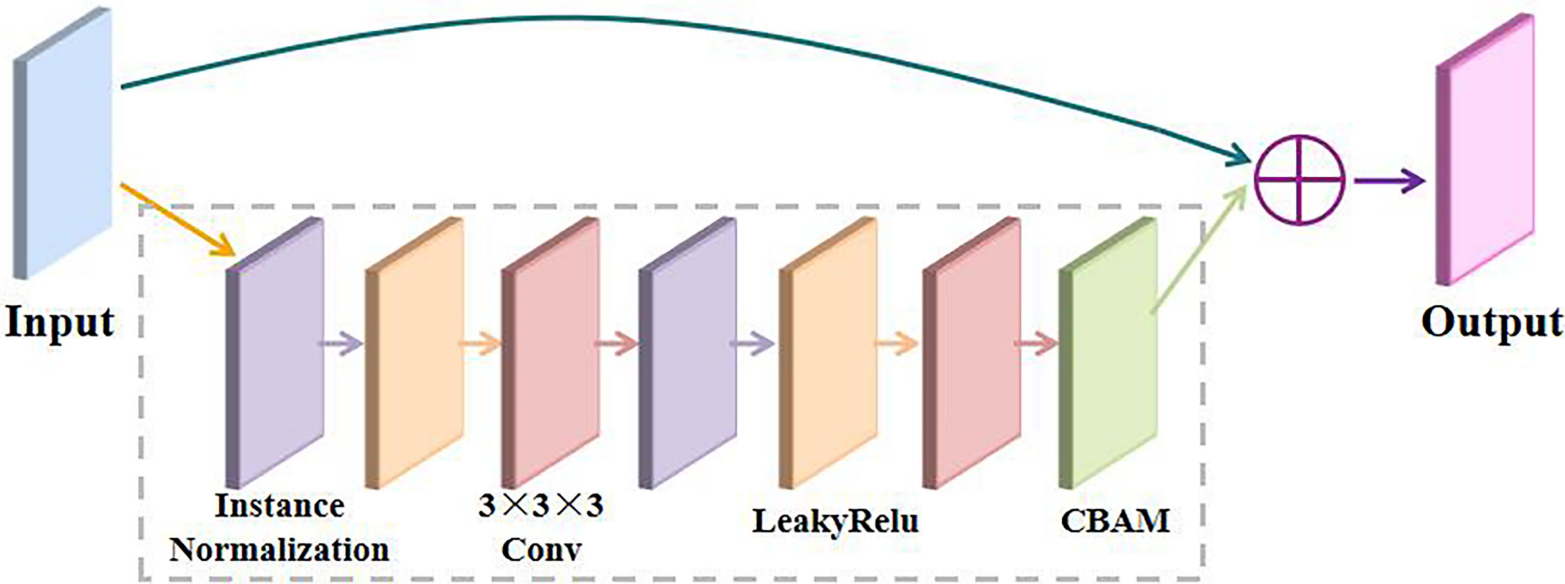
Structure of an RA block.

We propose a residual block with a 3D CBAM, namely, an RA block that is composed of two 3×3×3 convolutional layers for the pink module, two normalization layers for the purple module, two activation layers for the yellow module, and an attention layer for the green module, as illustrated in **Figure 3**. Batch normalization (BN) is sensitive to the batch size because the mean and variance are calculated on a single batch. The instance normalization (IN) operation is performed within a single sample and does not depend on the batch. The leaky rectified linear unit (LR) is a variant of ReLU with a variation in response to input fractions of less than 0, thereby mitigating the sparsity of ReLU and alleviating some of the problems of neuronal death that are caused by ReLU. Therefore, we substitute the instance normalization (IN) and leaky rectified linear unit (LR) functions for the popular batch normalization (BN) and rectified linear unit (ReLU) functions, respectively. The RA block effectively improves the extraction of image feature information by adding a CBAM after the final convolution layer of the residual block, thereby improving the segmentation accuracy of the network.

### DA Block

**Figure 4:**
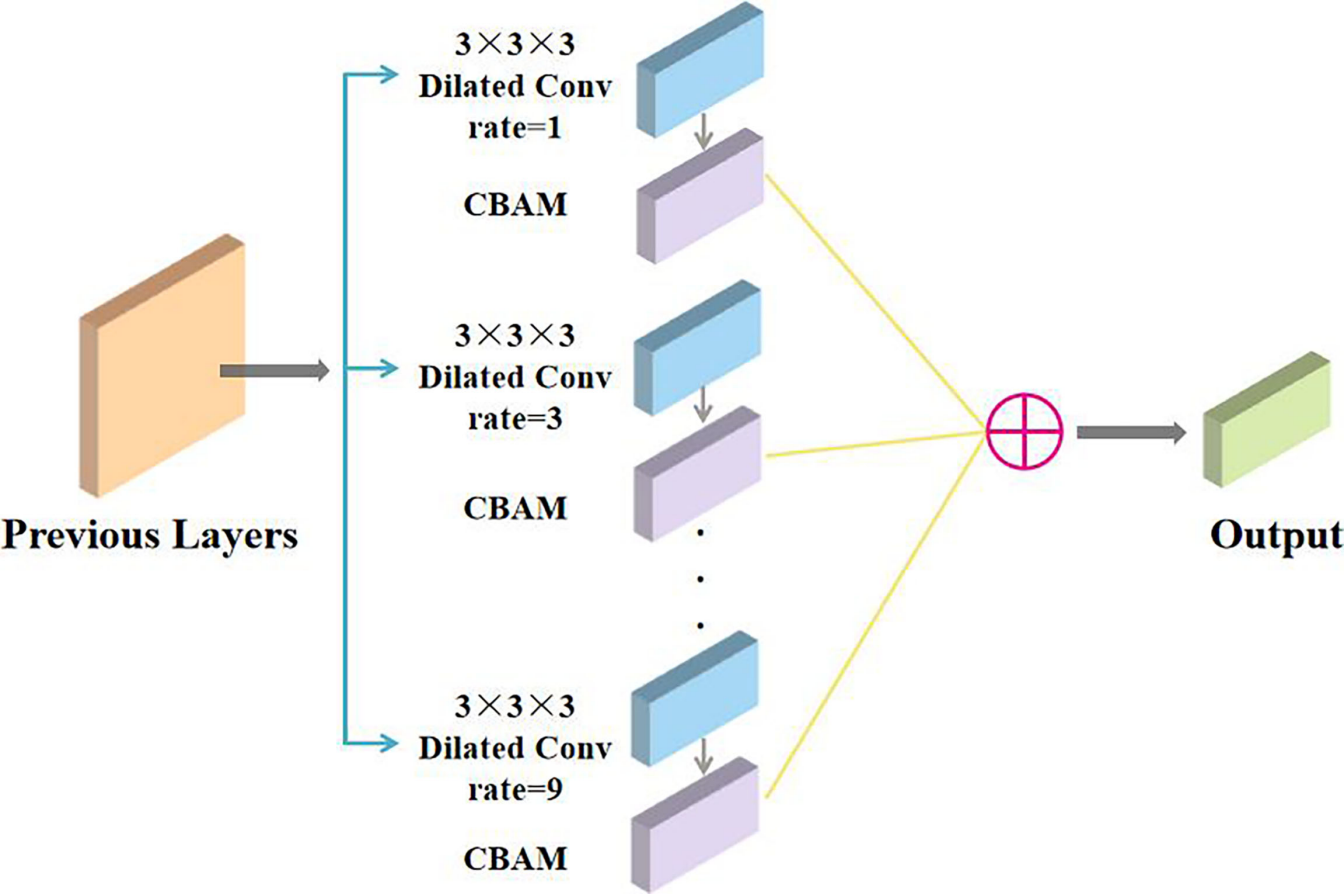
Structure of a DA block.

Our proposed DA block is shown as a deep blue block in **Figure 1**, which is composed of multiple green blocks of parallel 3×3×3 dilated convolution layers with various expansion rates and the orange CBAM blocks, and the multiple parallel feature maps are finally summed to obtain the output feature map of the purple block, as illustrated in **Figure 4**. Multiscale features are important for the segmentation of brain tumors. Therefore, we propose the DA block for efficiently obtaining feature maps of various sizes through levels of dilated convolutional layers in the spatial pyramid model.

### Dice Loss Function

The BraTS dataset is extremely unbalanced, and convolutional neural networks are very sensitive to unbalanced datasets. Therefore, we use the dice loss function to solve the problem. The dice function is expressed as follows:


(5) $$L=1-\frac{2*\sum_{n=1}^{N}p_{pred}*p_{true}}{\sum_{n=1}^{N}p_{pred}^{2}+p_{true}^{2}+\epsilon }$$

where P_pred_ is the decoder output, P_ture_ is the segmentation mask. where summation is voxel-wise, and ϵ is a small constant to avoid zero division.The process of RDAU-Net implementation is as follows. The 3D brain tumor data of four modalities are convolved twice by two 3×3×3 layers to increase the number of features in the initial filters. Then, the extracted feature maps are input into the RA block for feature extraction. The CBAM is used to exploit useful information of the input features. The feature maps that are extracted by the first RA block are downsampled by a convolutional layer with a step size of 2. After four rounds of convolution and downsampling, the extracted feature maps are input into the DA block for feature extraction with various feature sizes. The DA block combines multiple dilated convolutional layers in parallel and incorporates a CBAM dual attention mechanism behind each convolutional layer. This attention pyramid pooling module effectively obtains feature maps of various sizes through levels of expanded convolutional layers in the spatial pyramid model while extracting useful information on channels and spaces to increase the tumor segmentation accuracy. Then, the feature maps that are extracted from the DA block are upsampled by deconvolution. The upsampled feature maps are connected by a skip connection with the feature maps that have undergone feature weighting by the CBAM block in the corresponding layer in the encoder. Our proposed method inserts a CBAM block after each skip connection layer to improve the extraction of channel information and spatial information through tandem channel attention and spatial attention as a way to reduce the redundant information of low-level features. Attention is produced when the skip connection connects the feature maps that are extracted by the encoder directly to the corresponding layers of the decoder. After upsampling, the final prediction result of the network is output through the sigmoid function. Finally, an image of the same size as the input image is generated.

## Experiments

### Evaluation Indicators

We use the Dice coefficient, Hausdorff distance, sensitivity, and specificity evaluation metrics to evaluate our experimental results.

The Dice coefficient (Dice) is defined as:


(6) $$DSC=\frac{2\times TP}{FP+FN+2\times TP}$$

Sensitivity and specificity are defined as:


(7) $$\text{Sen}sitivity=\frac{TP}{TP+FN}$$

(8) $$Specificity=\frac{TP}{TP+FP}$$

where TP, FP, and FN indicate the true positive, false positive, and false negative values, respectively.

The Hausdorff distance indicates the maximum mismatch between the predicted edge of the tumor segmentation result and the ground-truth boundary.


(9) $$HD(T,S)=\max\{\sup_{x\in X}\inf_{y\in Y}d(x,y),\sup_{y\in Y}\inf_{x\in X}d(x,y)\}$$

where sup and inf denote the upper and lower boundaries, respectively, of the brain tumor region; x and y are points on the tumor surface, where *x* ∈ *T* and *y* ∈ *P*; and d() is the distance function.

### Experimental Details

The experiments are carried out on a workstation that is configured with an Intel^®^ Xeon(R) CPU E5-2620 v4 @ 2.10 GHz × 32 and equipped with two 12 GB TITAN Xp graphics cards. The proposed network is tested under the environment of TensorFlow-gpu==1.10.0^1^ and Keras==2.2.0^2^. For training, the input preprocessed image has an image block size of 4 × 128 × 128 × 128. Since the computational complexity of the 3D image block is too high, we set the batch size to 2. The experiments are performed using the Adam optimization method, and the initial learning rate is set to Lr_init_ = 1˙10^–4^. The learning rate is reduced by 50% after 15 epochs if the validation loss is not improving, and we regularize with an 12 weight decay of 10^–5^. The size of the output prediction segmentation result is also 128×128×128. Finally, we reshape the data to a size of 240 × 240 × 155 by using the Nilearn package^3^.

### Dataset

**Figure 5:**
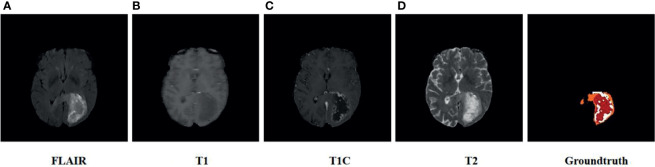
Schematic diagram of the BRATS 2018 training dataset. From left to right are **(A)** FLAIR, **(B)** T1, **(C)** T1-c, and **(D)** ground truth, where the colors of the ground truth represent categories of tumor segmentation: orange represents edematous regions, red represents enhancing tumors, and white represents necrotic and nonenhancing tumors.

Our proposed method is validated on the BraTS 2018 and 2019 (33–36) datasets. The BraTS 2018 dataset contains 285 glioma cases, which correspond to 210 HGG patients and 75 LGG patients. The validation set contains 66 MRs of patients with unknown tumor grade without real labels. The BraTS 2019 dataset contains 335 glioma cases, which correspond to 259 HGG patients and 76 LGG patients. The validation set contains 125 MRs of patients with unknown tumor grade without real labels. The dataset contains data of four modalities, namely, T1, T2, TIC, and FLAIR, and each MRI is a 3D image of size 240×240×155. The task of the segmentation challenge was to segment three tumor subregions: 1) the whole tumor (WT), 2) the tumor core (TC), and 3) the enhancing tumor (ET). **Figure 5** shows the modalities for a case in the BraTS 2018 training dataset and the ground truth.

### Data Preprocessing

MRI scans will often show intensity heterogeneity due to variations in the magnetic field. The variations in these mappings are called bias fields, and bias fields can cause problems for classifiers. We used N4ITK for bias field correction of the images, and N4ITK (36) is a modification of the N3 bias field correction method (37). Moreover, the images differ in terms of contrast among these four modes. Therefore, we normalize using the z-score method, namely, we subtract the intensity of each pixel from the average intensity of all pixels in each multimodal image and divide by the standard deviation, while GT is not normalized. Finally, we change the size from 240 × 240× 155 to 128 × 128 × 128 to reduce the number of parameters of the network.

### Ablation Study

To determine whether the CBAM dual attention module is effective in enhancing the segmentation performance of the network, we perform ablation experiments.

These experiments are conducted with and without the use of the attention module. The scores for the four evaluation metrics on the datasets of the BraTS 2018 and 2019 challenges are obtained separately. As presented in **Table 1**, the Dice coefficient and Hausdorff distance of the network in which the dual attention mechanism is utilized improved across the board on both the BraTS 2018 and 2019 challenge datasets, especially the Dice score of WT, which improved by 1% on both datasets, and the Hausdorff distance of ET, which decreased by 0.2 mm and 0.11 mm, respectively. In addition, the sensitivity of the model in which the attention mechanism is utilized improved by 0.5% and 0.2%, respectively, on ET. In general, adding CBAMs to the network can effectively improve the performance of the network.

**Table 1:** Segmentation results of our proposed network with and without attention mechanism on BraTS 2019 validation set using Dice, Hausdorff distance, specificity, and sensitivity metrics.

Models	Hausdorff (mm) ↓			Dice ↑			Specificity ↑			Sensitivity ↑		
	WT	TC	ET	WT	TC	ET	WT	TC	ET	WT	TC	ET
RDU-Net (without CBAM)	6.9705	6.1778	3.0002	0.8895	0.9018	0.8222	0.9894	0.9969	0.9958	0.9169	0.9031	0.8714
RDAU-Net	6.8692	6.0015	2.9980	0.9001	0.9061	0.8311	0.9892	0.9975	0.9966	0.9172	0.9028	0.8730

Results are specified for WT, whole tumor; ET, enhancing tumor; and TC, tumor core.

**Figure 6:**
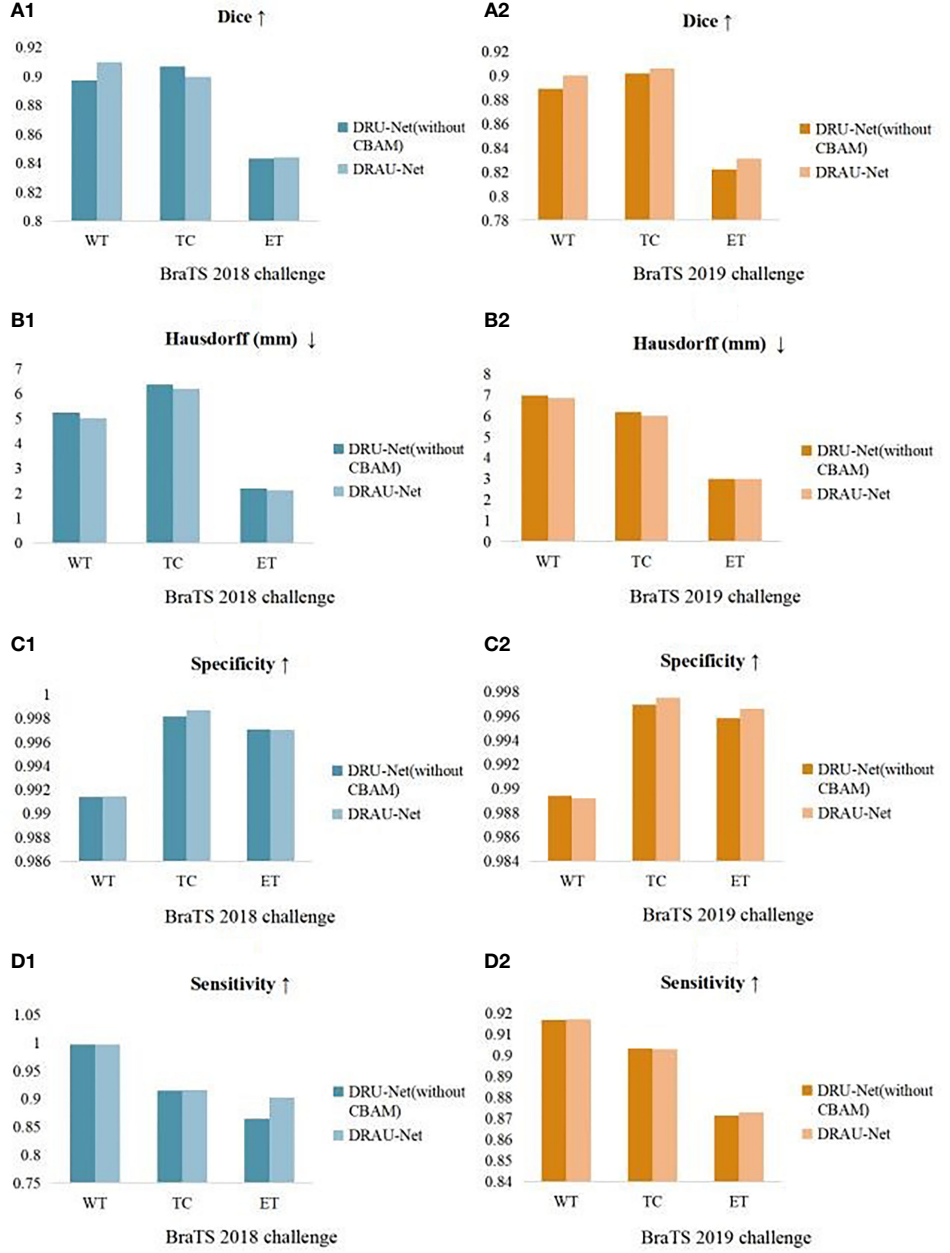
Histograms of the considered evaluation metrics on the datasets of the BraTS 2018 and 2019 challenges. **(A–D)**: Dice, Hausdorff (mm), specificity, and sensitivity. Blue indicates the BraTS 2018 challenge dataset and orange indicates the BraTS 2019 challenge dataset.

Histograms of various evaluation metrics on the datasets of the BraTS 2018 and 2019 challenges are presented in **Figure 6**. According to these results, inclusion of the CBAM in the network can effectively improve the segmentation accuracy of the model.

**Figure 7:**
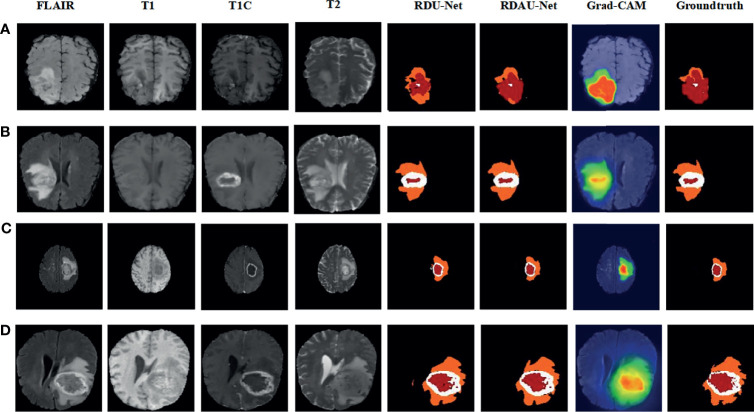
Segmentation results of the proposed method and RDU-Net (without attention) on the BRATS 2018 datasets, the Grad-CAM result and the ground truths. Rows **(A–D)**: Brats18_TCIA01_387_1_93, Brats18_TCIA01_231_1_83, Brats18_CBICA_APR_1_105, Brats18_CBICA_AUN_1_81, where 105, 101, 78, and 73 represent the 105th, 101st, 78th, 73rd slices, respectively, of the MRI data. The colors represent the following categories: 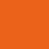 edema, 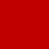 necrosis, and □ enhancing core.

**Figure 7** shows the visual segmentation results of our proposed approach on the BraTS 2018 challenge data training set. From **Figure 7**, we find that our network model can segment various regions of the tumor, especially the ET parts of the tumor, but there are segmentation errors in small places compared with the ground truth. In summary, by comparing our segmentation results with those of RDU-Net (without attention) and the ground truth, we find that our proposed model obtains satisfactory segmentation results with the RA block and the DA block.

### Results on the BraTS Challenge Training Data

**Figure 8:**
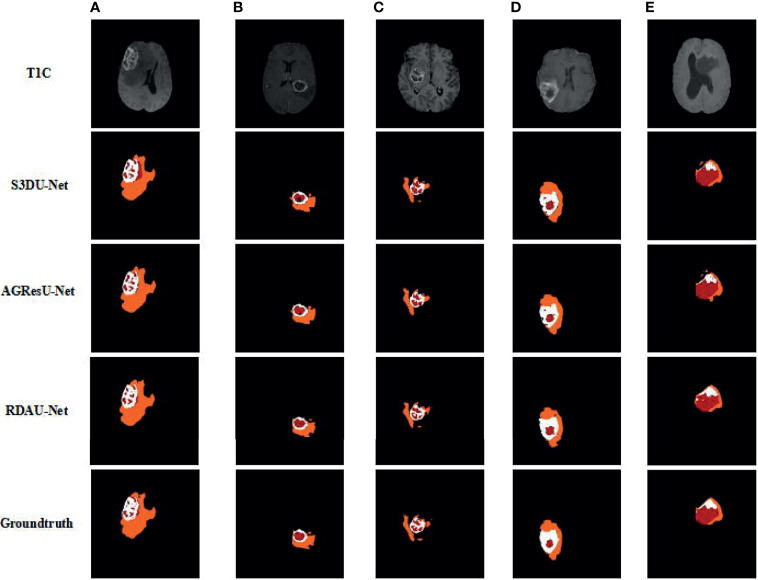
Results of brain tumor segmentation in MRI images using the proposed approach and the ground truths. Columns **(A–E)** correspond to cases in the Brats 2018 challenge dataset: Brats18_2013_12_1_89, Brats18_CBICA_AQV_1_87, Brats18_TCIA02_117_1_69, Brats18_TCIA04_361_1_90, and Brats18_TCIA05_444_1_84, where 89, 87, 69, 90, and 84 represent the 89th, 87th, 69th, 90th, and 84th slices, respectively, of the MRI data. The colors represent the following categories: 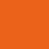 edema, 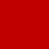 necrosis, and □ enhancing core.

We compare the segmentation performance of our approach with those of other typical deep network methods, as shown in **Figure 8**, on the BraTS 2018 challenge training dataset. The original data in **Figure 8** from top to bottom are T1-c; the segmentation results of the S3DU-Net (38) model, the AGResU-Net (39) model, and our RDAU-Net method; and the ground truths. By comparing these methods in **Figure 8**, we observe that the segmentation results of our proposed method, namely, RDAU-Net, are the closest to the ground truth on WT, TC, and ET and that the segmentation results are significantly better than those of the remaining two methods.

### Comparison With State-of-the-Art Methods

#### Performance Comparison on the BraTS 2018 Challenge Dataset

**Figure 9:**
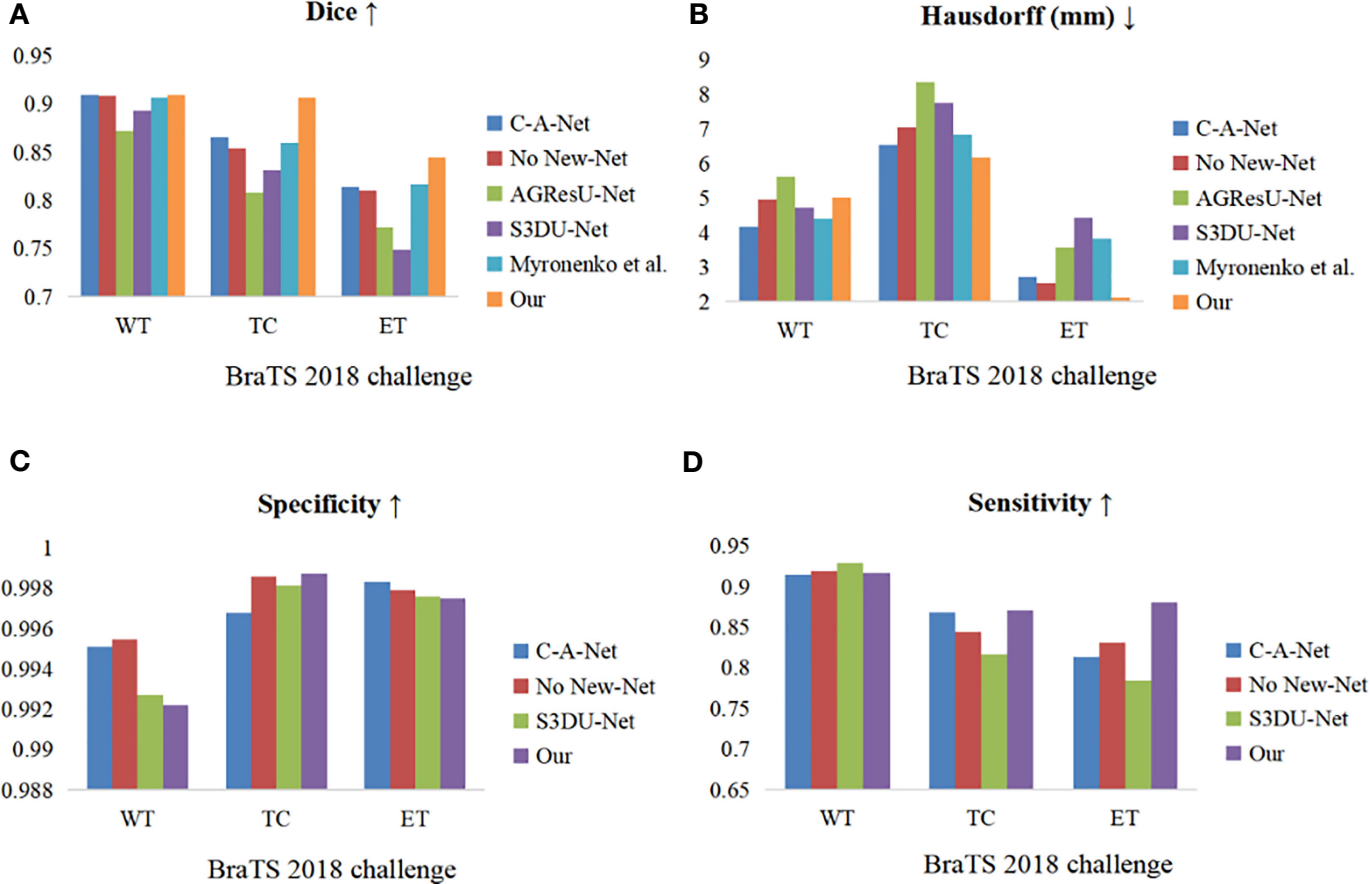
Comparative histograms of evaluation indicators for four methods on the BraTS 2018 validation set. **(A–D)** Dice, Hausdorff (mm), specificity, and sensitivity. The colors correspond to the methods.

The first-place winner of the Multimodal Brain Tumor Segmentation Challenge (BraTS) 2018 was Myronenko et al., who trained their model using large image blocks with a size of 160×192×128. As presented in **Table 2**, the Myronenko et al. (40) method has a higher overall Dice score than other mainstream methods, but our method outperforms Myronenko’s method in terms of Dice score by 0.26%, 4.7%, and 3% for WT, TC, and ET, respectively. Our method also outperforms Myronenko’s method in terms of Hausdorff distance, which is reduced by 0.7 mm and 1.7 mm for TC and ET, respectively. The No New-Net (41) method won second place with only a few minor changes to U-Net. As presented in **Table 2**, our method outperforms their method overall in segmentation. The Dice scores on TC and ET are 5% and 3.5% higher than theirs, respectively; the Hausdorff distances on TC and ET are 0.9 mm and 0.4 mm shorter than theirs, respectively; and the sensitivity scores on TC and ET are 3% and 5% better than theirs, respectively. C-A-Net (42) is a single-channel multitask network that combines multiple CNN structures. According to **Table 2**, our network outperforms the C-A-Net method on all metrics except the Hausdorff distance score on WT, and the Dice scores on TC, and ET are improved by 5%, and 3.5%, respectively. Our sensitivity score on ET is nearly 5% higher than that of the C-A-Net method. The AGResU-Net method (39) integrates the residual module and attention gates in the original U-Net. As presented in **Table 2**, our method outperforms the network on all metrics, especially the Hausdorff distance on TC: our method obtains a Hausdorff distance that is 2.1 mm shorter than that obtained by the AGResU-Net method, and our method obtains Dice scores that are improved by 9% and 7% on TC and ET, respectively. The S3DU-Net method (38) is a module in which the convolutional block in U-Net is changed to a 3D convolution with three parallel branches. It scores well on the sensitivity metric on WT, but our network outperforms that network overall. Compared with the S3DU-Net method, our method improves the Dice score by 7% and 9% on TC and ET, respectively; reduces the Hausdorff distance by 1.5 mm and 2.3 mm on TC and ET, respectively; and improves the sensitivity by 5% and 9% on TC and ET, respectively. The histograms in **Figure 9** compare several methods in terms of various metrics. According to the comparison in **Figure 9**, our method is highly competitive.

**Table 2:** Comparison of performance between our approach and the state-of-the-art methods on BraTS 2018 validation set using Dice, Hausdorff distance, specificity, and sensitivity metrics.

Models	Hausdorff (mm) ↓			Dice ↑			Specificity ↑			Sensitivity ↑		
	WT	TC	ET	WT	TC	ET	WT	TC	ET	WT	TC	ET
Myronenko (40)	4.41	6.84	3.82	0.9068	0.8602	0.8173	–	–	–	–	–	–
No New-Net (41)	4.97	7.04	2.54	0.9083	0.8544	0.8101	**0.9955**	0.9986	0.9979	0.9187	0.8444	0.8312
C-A-Net (42)	**4.1724**	6.5445	2.7162	**0.9095**	0.8651	0.8136	0.9951	0.9968	**0.9983**	0.9142	0.8683	0.8135
AGResU-Net (39)	5.62	8.36	3.57	0.872	0.808	0.772	–	–	–	–	–	–
S3DU-Net (38)	4.7165	7.7478	4.4321	0.8935	0.8309	0.7493	0.9927	0.9981	0.9976	**0.9290**	0.8161	0.7849
Our	5.0206	**6.1829**	**2.1260**	0.9094	**0.9071**	**0.8446**	0.9922	**0.9987**	0.9975	0.9161	**0.8699**	**0.8805**

Results are specified for WT, whole tumor; ET, enhancing tumor; and TC, tumor core. The results are marked in bold.

#### Performance Comparison on the BraTS 2019 Challenge Dataset

**Figure 10:**
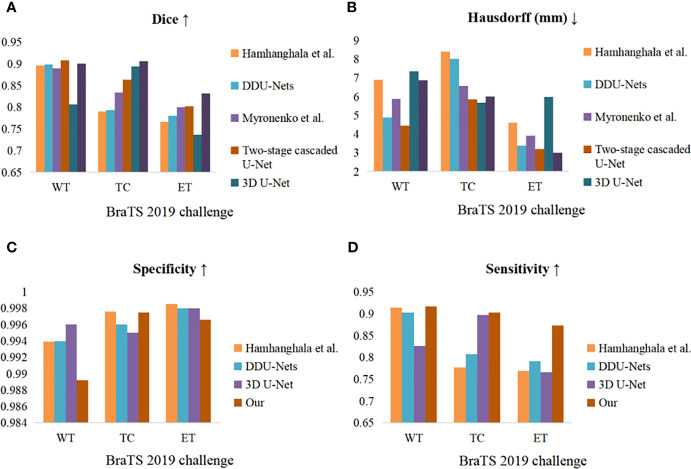
Comparative histograms of evaluation indicators for four methods on the BraTS 2019 validation set. **(A–D)** Dice, Hausdorff (mm), specificity, and sensitivity. The colors correspond to the methods.

For the BraTS 2019 challenge dataset, RDAU-Net still achieved the best results in terms of the Hausdorff distance metric in the tumor core region and the Dice coefficients of TC and ET and performed very competitively in terms of other metrics. The two-stage cascaded U-Net (43) won first place in the BraTS 2019 challenge. Compared with the champion team, the Dice scores and the sensitivity scores on ET and TC of our method are almost 3% and 4.3% higher and 7% and 4% higher, respectively. The Hausdorff distance of the ET subregion that was obtained using our method is approximately 0.2 mm shorter. Hamhanghala et al. (44) used generative adversarial networks to expand the data. Although the training sample was expanded with synthetic data, this approach did not yield more prominent results. DDU-Nets (45) contain three models of distributed dense connectivity. DDU-Nets are not particularly effective overall, although they slightly outperform our method in terms of Hausdorff distance scores on WT and ET. Myronenko et al. (46) further improved the loss function based on 2018 by introducing the focal loss and using eight 32 G video cards for training. As presented in **Table 3**, our network still outperforms Myronenko et al.’s method on all other metrics, except for a slightly lower Hausdorff distance score on WT than their method. The 3D U-Net (47) is designed mainly to handle block diagrams. From **Table 3**, our method outperforms the 3D U-Net method on all metrics except for the Dice score on TC and the specificity scores on WT and ET, which are slightly lower than those of the 3D U-Net method. The histograms in **Figure 10** clearly compare the considered methods in terms of various metrics. According to the comparison, our method is still very competitive, even on the newer dataset.

**Table 3:** Comparison of performance between our approach and the state-of-the-art methods on BraTS 2019 validation set using Dice, Hausdorff distance, specificity, and sensitivity metrics.

Models	Hausdorff (mm) ↓			Dice ↑			Specificity ↑			Sensitivity ↑		
	WT	TC	ET	WT	TC	ET	WT	TC	ET	WT	TC	ET
Two-stage cascaded U-Net (43)	**4.4438**	5.8620	3.2055	**0.9082**	0.8632	0.8020	0.9943	0.9974	0.9984	0.9237	0.8622	0.8038
Hamhanghala et al. (44)	6.9	8.4	4.6	0.8965	0.7901	0.7665	0.9939	0.9976	**0.9985**	0.9132	0.7771	0.7688
DDU-Nets46]	4.874	8.013	3.376	0.898	0.793	0.780	0.994	0.996	0.998	0.903	0.808	0.791
Myronenko et al. (46)	5.89	6.562	3.921	0.8900	0.8340	0.8000	–	–	–	–	–	–
3D U-Net (47)	7.357	**5.667**	5.994	0.807	0.894	0.737	**0.996**	0.995	0.998	0.826	0.897	0.766
Our	6.8692	6.0015	**2.9980**	0.9001	**0.9061**	**0.8311**	0.9892	**0.9975**	0.9966	**0.9172**	**0.9028**	**0.8730**

Results are specified for WT, whole tumor; ET, enhancing tumor; and TC, tumor core. The results are marked in bold.

## Conclusions

We propose a new method, namely, RDAU-Net that is based on an improved ResU-Net for brain tumor segmentation in MRI. We add DA blocks to expand the receptive field and obtain image information of various sizes and insert a CBAM block after each skip connection layer to improve the extraction of channel information and spatial information to reduce the redundant information of low-level features. By conducting experiments on the BraTS 2018 and BraTS 2019 datasets, we find that the use of an RA block instead of a convolutional layer, the inclusion of a DA block in the network, and the insertion of 3D CBAM blocks can effectively improve the performance of the network. RDAU-Net has more obvious advantages than the SOTA method. However, the performance of the method in the WT region still has substantial room for improvement, and we hope to solve this problem through postprocessing of the network. In conclusion, the method has greater advantages in segmenting subregions of brain tumors and can be effectively applied to clinical research.

## Data Availability Statement

Publicly available datasets were analyzed in this study. This data can be found here: https://doi.org/10.7937/K9/TCIA.2017.KLXWJJ1Q.

## Author Contributions

JW provided guidance on the content of the article as well as the writing of the article. ZY writes articles and conducts experiments. ZL and JR completed the work of collecting references. YZ provided guidance on medical knowledge and medical image knowledge. GY provided guidance on the experiments done in the article. All authors are approved for publication.

## Funding

This work was jointly supported by the Shandong University Science and technology project (No.J17KA0 82, No. J16LN21), the National Natural Science Foundation of China (No.61401259), the China Postdoctoral Science Foundation (No.2015M582128).

## Conflict of Interest

The authors declare that the research was conducted in the absence of any commercial or financial relationships that could be construed as a potential conflict of interest.

## Publisher’s Note

All claims expressed in this article are solely those of the authors and do not necessarily represent those of their affiliated organizations, or those of the publisher, the editors and the reviewers. Any product that may be evaluated in this article, or claim that may be made by its manufacturer, is not guaranteed or endorsed by the publisher.

## Footnotes

1 https://github.com/TensorFlow/TensorFlow

2 https://github.com/keras-team/keras

3 https://github.com/nilearn/nilearn
